# Shared decision making between patient and GP about referrals from primary care: Does gatekeeping make a difference?

**DOI:** 10.1371/journal.pone.0198729

**Published:** 2018-06-11

**Authors:** Alexandru M. Rotar, Michael J. Van Den Berg, Willemijn Schäfer, Dionne S. Kringos, Niek S. Klazinga

**Affiliations:** 1 Department of Public Health, Academic Medical Center, University of Amsterdam, Amsterdam Public Health Research Institute, Amsterdam, The Netherlands; 2 NIVEL (Netherlands Institute for Health Services Research), Utrecht, The Netherlands; Universidad del Desarrollo, CHILE

## Abstract

Primary care faces challenging times in many countries, mainly caused by an ageing population. The GPs’ role to match patients’ demand with medical need becomes increasingly complex with the growing multiple conditions population. Shared decision-making (SDM) is recognized as ideal to the treatment decision making process. Understanding GPs’ perception on SDM about patient referrals and whether patients’ preferences are considered, becomes increasingly important for improving health outcomes and patient satisfaction. This study aims to 1) understand whether countries vary in how GPs perceive SDM, in patients’ referral, 2) describe to what extent SDM in GPs’ referrals differ between gatekeeping and non-gatekeeping systems, and 3) identify what factors GPs consider when referring to specialists and describing how this differs between gatekeeping and non-gatekeeping systems. Data were collected between October 2011 and December 2013 in 32 countries through the QUALICOPC study (Quality and Costs of Primary Care in Europe). The first question was answered by assessing GPs’ perception on who takes the referral decision. For the second question, a multilevel logistic model was applied. For the third question we analysed the GPs’ responses on what patient logistics and need arguments they consider in the referral process. We found: 1) variation in GPs reported SDM– 90% to 35%, 2) a negative correlation between gatekeeper systems and SDM—however, some countries strongly deviate and 3) GPs in gatekeeper systems more often consider patient interests, whereas in non-gatekeeping countries the GP’s value more own experience with specialists and benchmarking information. Our findings imply that GPs in gatekeeper systems seem to be less inclined to SDM than GPs in a non-gatekeeping system. The relation between gatekeeping/non-gatekeeping and SDM is not straightforward. A more contextualized approach is needed to understand the relation between gatekeeping as a system design feature and its relation with and/or impact on SDM.

## Introduction

Primary care is facing challenging times in many countries, mainly caused by an ageing population and increase in the burden of disease. The delivery of care requires a new approach built around the fundamentals of primary care, particularly continuity and care coordination.[1] The need to move to more efficient and patient centric primary care, pressures decision-makers for reform. In western countries, primary care is often the first contact point between the public and the health care system. Strong primary care is often associated with the gatekeeping position of GPs.[2] Strengthening primary care increases incentives for the gatekeeping role of GPs. Most often, GPs are better informed on options for treatments by medical specialists than patients.[3] By performing the gatekeeping role, GPs mitigate supplier-induced demand by handling the asymmetry of information between patients and specialists, limiting the possibility of the specialists to create their own market.

Economic theory places GPs’ gatekeeping role as a restriction on the demand side. In healthcare, patient demand is matched with the medical needs as perceived by the health care professional. Therefore, the GPs’ role is to match the demand of the patients with their medical need, which becomes increasingly complex with the growing number of persons with multiple conditions. By many, the gatekeeping function is considered to promote coordination of care, and is perceived to work well when the GP-patient relationship is based on trust. It is also perceived as a tool for rationing, by limiting access to specialists through regulating referrals. [2][3][4]

For decades, countries like the U.K. and the Netherlands have had a primary care system with a gatekeeping role.[5][6] In the U.K., its introduction was intended to be a response to specialists’ shortage by slowing down the rate of referral to help regulate waiting times for secondary care, while it was seen also as a tool to control costs.[7] However, it is unclear if the intended effects on decreased costs occurred. There is evidence on both sides, showing associations with increases and decreases in healthcare utilization and costs.[8][9]

Gatekeeping has also showed a positive relationship with a reduction in the adverse effects of overtreatment.[10] However, gatekeeping may have “unexpected, serious side effects”, as associations were found with lower survival rates in oncology, potentially caused by delayed diagnosis.[11] There are stakeholders who have been lobbying actively for abolition of gatekeeping believing it limits patient choice. More recently, it has been suggested, “gatekeeping negates the person centered model, patient choice, and shared decision making”.[12][13]

Despite its critiques and confounding evidence, strong primary care systems, with a gatekeeping role in place, have shown associations with improved population health outcomes, reduced socio-economic inequalities, fewer avoidable hospitalizations, and more continuity as perceived by patients.[14][15] Since the early 1990’s, Central Eastern European countries reformed their health care systems by introducing new concepts, such as gatekeeping. However, gatekeeping came along with a reputation as a bureaucratic hurdle to specialized medical services and having generalist doctors with less medical knowledge making key decisions.[10] Irrespective of the GPs’ gatekeeping role intensity in different countries, patients’ involvement in healthcare has been widely recognized by the medical community [16] and needs to be aligned with strategies aimed at promoting efficiency.

In the past decades, SDM has been recognized as an ideal for the treatment decision making process. [17] SDM seems to be of benefit for disadvantage groups, improve cognitive outcomes and quality of life.[18][19][20] Still, general outcomes are yet undetermined. Nevertheless, understanding the GPs’ perception on shared decision making about patient referrals, and whether patients’ preferences are considered, becomes increasingly important. It is also unknown to what extent logistical factors and need influence GPs’ professional behavior with regard to referrals. Additionally, there is no evidence for any association between shared decision-making, the referral practice and the typology of the primary care system, gatekeeping system vs. non-gatekeeping system. Our aim is to investigate these elements. More specifically, our study addresses three research questions, by analyzing 32 countries with different historical background on their healthcare systems typology:

Do countries vary in how GPs perceive shared decision-making (SDM), in deciding upon patients’ referral?
To what extent does shared-decision making in GPs’ referrals differ between gatekeeping and non-gatekeeping systems?
What factors do GPs consider when referring to a medical specialist and how does this differ between gatekeeping and non-gate keeping systems?

## Materials and methods

To address the research questions we used the GPs survey data from the cross-sectional QUALICOPC study (Quality and Costs of Primary Care in Europe), which included 31 European countries and Australia, Canada and New Zealand. Data collection took place between October 2011 and December 2013. The aim for each country was to draw a nationally representative sample of GPs with one GP per practice.[21] The GP questionnaire was completed by 7,183 GPs. Details about the study protocol, questionnaire development and data collection, have been published elsewhere.[21][22][23] The study is written within the aims of the QUALICOPC study and its ethical approval. Ethical review was conducted in accordance with the legal requirements in each country. See the S1 Table for an overview of names of ethics committees by country.

Greece and Finland were excluded from our analysis after validating the primary findings with the country coordinators. In Greece, there was a translation error in the question to be analyzed, and in Finland, at the time of the survey, there were some developments in the health care system that impacted the reliability of the physicians’ answers to the questions related to this topic.

To answer the first research question we used the GP’s reported perception on who makes the decision in case of referrals. The participating GPs were asked through the survey to respond 1) if they make the decision, 2) if the patient does or 3) if it is a shared decision.

For the second question, a multilevel logistic model was applied. The response to the question on who decides in case of a referral was dichotomized into 1 (it is a shared decision) and 0 (either the patient of the doctor). We call this variable ‘shared decision making’ (SDM). The model has two levels, with GPs (level 1) clustered into countries (level 2). In step-1, the variable ‘gatekeeping’ (0 for no, 1 for yes) was added to the model. In step 2, controlling variables were added to the model: age and sex of the GP and the practice location—big (inner)city, suburbs, (small) town, mixed urban-rural and rural.

For the third research question we analysed the GPs responses on what patient logistics and need arguments they consider in the referral process, including: 1) the patient’s preference where to go, 2) the travel distance for the patient, 3) the physician’s previous experiences with the medical specialist, 4) comparative performance information on medical specialists, 5) waiting time for the patient and 6) costs for the patient. Each of the 6 items had three answer options: *always*, *sometimes and never*. Our analysis focused on the *“always”* since if considering *“always”* and *“sometimes”* as a positive answer, there were almost no differences between countries and between the aspects they take into consideration (see annex 1). We compiled the data based on the responses and benchmarked countries 1) against each other, 2) against the total weighted average, 3) the gatekeeping systems against the total weighted average of the gatekeeping systems and 4) the non-gatekeeping systems against the total weighted average of the non-gatekeeping. To classify whether primary care systems have a gatekeeping system in place or not, the QUALICOPC, OECD and PHAMEU data bases were examined. [24][25] For reasons of consistency the taxonomy of QUALICOPC data was used.

## Results

**Fig 1:**
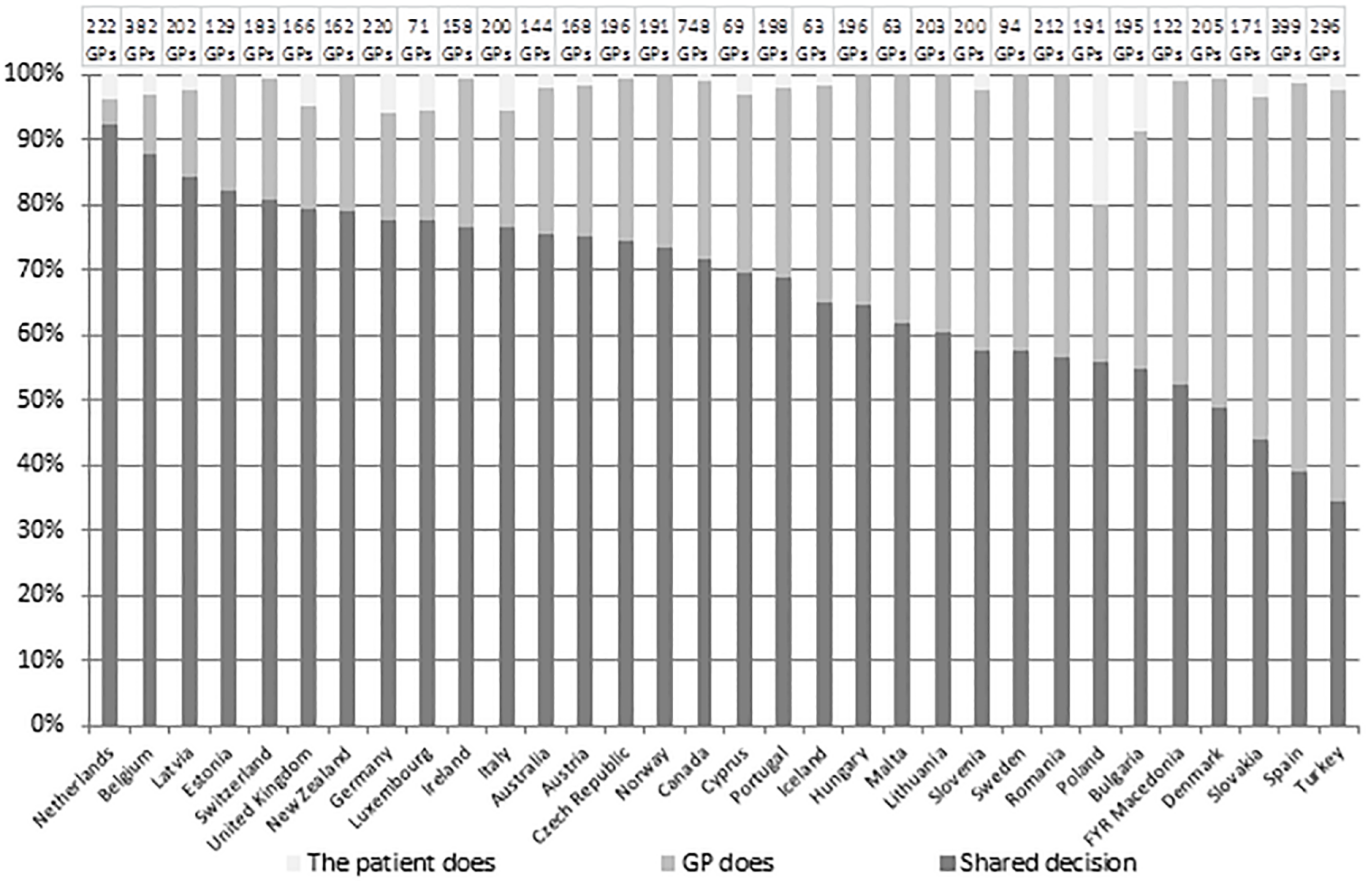
Answer to the question: In case of referral, who usually decides about where the patient is referred?

Fig 1 shows the reported levels of referral practice across 32 countries. “SDM” is reported to be most common (levels higher than 50%) in 28 countries. The values range from 92.3% (the Netherlands) to 52.5% (FYR Macedonia). GP-based decision is most common in 4 countries:—Denmark (50.7%), Slovakia (52.6%), Spain (59.9%) and Turkey (63.2%). Patients being the main decision-taker is reported far less frequently. In 25 countries the values range low, from 0.5% (the Czech Republic and Denmark) to 8.7% (Bulgaria) and 19.9% (Poland), while in the remaining 7 countries, the patient never makes the decision.

The results of our logistic regression analysis are shown in Table 1. The first model (column 2) shows a negative odds ratio for gate keeping, suggesting that GPs in gatekeeping systems are less likely to make shared decisions. This negative relation remains after controlling for a number of variables, such as female doctors and practice location (model 2). Female doctors and those in rural areas are more likely to report that they make shared decisions.

**Table 1:** Results logistic regression multilevel analyses association between the shared decision making (SDM) levels and gate keeping system.

N^i^ = 32; Nj = 6572	Odds ratio	SE	Odds ratio	SE
Intercept	2.387	0.035	1.520	0.158
Gatekeeping (baseline NO)	0.865	0.053	0.859	0.055
Age			1.002	0.002
Gender—male/female (baseline—MALE)			1.208	0.055
Practice location				
Suburbs (baseline—Big city)			1.342	0.089
Small town (baseline—Big city)			1.335	0.072
Mixed urban-rural (baseline—Big city)			1.589	0.083
Rural (baseline—Big city)			1.839	0.085

Table 2 shows the amounts to which different considerations are taken into account when referring a patient. Patient preference (48.5%) and previous experience with specialists (59.1%) are reported to be the most important factors affecting GPs decision on referrals. There is, however, a high variation between countries on the importance of patients’ preference, ranging from 15% to 81%. Only a few countries stand out for using benchmark information (Czech Republic, Bulgaria and Romania) while this is virtually not-used in some others (Iceland and Norway). In some cases very practical reasons play a role. For instance, travel time which is mainly an issue in large geographically spread countries such as Australia and Canada.

**Table 2:** Answer to the question: In case of referral, to what extent do you take into account the following considerations?

Country	Total no. of GPs	Always takes into account												
		patient’s preference where to go		travel distance for the patient		own previous experience with the specialist		comparative performance information on specialists		waiting time for the patient		costs for the patient		gatekeeping system (GK) QUALICOPC
Austria	177	85	48.0%	97	54.8%	137	77.4%	37	20.9%	20	11.3%	68	38.4%	NO
Belgium	384	283	73.7%	103	26.8%	301	78.4%	100	26.0%	60	15.6%	64	16.7%	NO
Cyprus	70	26	37.1%	17	24.3%	21	30.0%	15	21.4%	14	20.0%	22	31.4%	NO
Czech Republic	207	67	32.4%	38	18.4%	169	81.6%	145	70.0%	29	14.0%	18	8.7%	NO
Denmark	206	89	43.2%	49	23.8%	135	65.5%	92	44.7%	51	24.8%	52	25.2%	NO
Germany	234	103	44.0%	131	56.0%	172	73.5%	33	14.1%	31	13.2%	31	13.2%	NO
Iceland	74	32	43.2%	18	24.3%	44	59.5%	3	4.1%	6	8.1%	12	16.2%	NO
Luxembourg	73	54	74.0%	16	21.9%	59	80.8%	27	37.0%	10	13.7%	5	6.8%	NO
Malta	65	41	63.1%	11	16.9%	32	49.2%	20	30.8%	28	43.1%	34	52.3%	NO
Poland	192	104	54.2%	87	45.3%	92	47.9%	60	31.3%	102	53.1%	91	47.4%	NO
Slovakia	185	28	15.1%	36	19.5%	86	46.5%	41	22.2%	35	18.9%	32	17.3%	NO
Switzerland	189	103	54.5%	81	42.9%	167	88.4%	34	18.0%	30	15.9%	27	14.3%	NO
Turkey	297	64	21.5%	99	33.3%	107	36.0%	40	13.5%	73	24.6%	132	44.4%	NO
FYR Macedonia	122	30	24.6%	33	27.0%	66	54.1%	24	19.7%	45	36.9%	58	47.5%	NO
Bulgaria	199	104	52.3%	61	30.7%	136	68.3%	112	56.3%	48	24.1%	81	40.7%	YES
Estonia	129	71	55.0%	36	27.9%	75	58.1%	11	8.5%	38	29.5%	35	27.1%	YES
Hungary	205	81	39.5%	89	43.4%	118	57.6%	48	23.4%	81	39.5%	98	47.8%	YES
Ireland	162	106	65.4%	71	43.8%	108	66.7%	31	19.1%	70	43.2%	81	50.0%	YES
Italy	201	72	35.8%	39	19.4%	129	64.2%	63	31.3%	61	30.3%	77	38.3%	YES
Latvia	206	114	55.3%	76	36.9%	139	67.5%	70	34.0%	98	47.6%	83	40.3%	YES
Lithuania	216	79	36.6%	19	8.8%	68	31.5%	36	16.7%	40	18.5%	19	8.8%	YES
Netherlands	226	195	86.3%	103	45.6%	114	50.4%	18	8.0%	43	19.0%	26	11.5%	YES
Norway	195	67	34.4%	48	24.6%	84	43.1%	13	6.7%	58	29.7%	56	28.7%	YES
Portugal	205	80	39.0%	77	37.6%	72	35.1%	55	26.8%	81	39.5%	103	50.2%	YES
Romania	214	84	39.3%	60	28.0%	115	53.7%	113	52.8%	66	30.8%	107	50.0%	YES
Slovenia	203	41	20.2%	36	17.7%	80	39.4%	37	18.2%	65	32.0%	32	15.8%	YES
Spain	409	84	20.5%	96	23.5%	246	60.1%	153	37.4%	96	23.5%	74	18.1%	YES
Sweden	95	39	41.1%	23	24.2%	49	51.6%	14	14.7%	33	34.7%	22	23.2%	YES
United Kingdom	167	126	75.4%	88	52.7%	62	37.1%	22	13.2%	60	35.9%	32	19.2%	YES
Australia	149	121	81.2%	97	65.1%	123	82.6%	41	27.5%	57	38.3%	82	55.0%	YES
Canada	754	501	66.4%	425	56.4%	484	64.2%	112	14.9%	350	46.4%	302	40.1%	YES
New Zealand	162	116	71.6%	65	40.1%	92	56.8%	22	13.6%	62	38.3%	108	66.7%	YES
total	6572	3190	48.5%	2325	35.4%	3882	59.1%	1642	25.0%	1941	29.5%	2064	31.4%	
total GK	4097	2081	50.8%	1509	36.8%	2294	56.0%	971	23.7%	1407	34.3%	1418	34.6%	
total non GK	2475	1109	44.8%	816	33.0%	1588	64.2%	671	27.1%	534	21.6%	646	26.1%	
Diff			6.0%		3.9%		-8.2%		-3.4%		12.8%		8.5%	
p			<0.005		<0.005		<0.005		<0.005		<0.005		<0.005	

White denotes 0–25%, red denotes >25–50%, yellow denotes >50–75%, green denotes >75–100%.

GPs in countries with gatekeeping systems in place are more likely to always take into account patients’ preference, travel distance waiting time and costs, while the GPs from countries with no gatekeeping in place are more likely to consider their previous experience with a specialist and comparative performance information. The “p” value is <0.005 for all items.

## Discussion

The first question of this study was whether countries vary to the extent GPs report shared decision-making (SDM), in deciding upon patients’ referral. Although GPs reported that SDM is the most common practice in 20 of the 32 countries, the results showed a strong variation between countries, ranging from over 90% to 35%. When decisions on referrals are not a shared decision, in most cases it is the GP who decides. Patients being the main decision-taker was reported rarely. Only one country, Poland, stands out for a particularly high percentage of decisions made by patients. This finding was explained by the Polish country coordinator based on the patients’ legal right of a “free choice”, i.e. GPs are not allowed to have the name of a specific specialist on the referral letter (personal communication with Prof. Dr. Adam Windak on June 8^th^, 2017).

To what extent does SDM in GPs referrals differ between gatekeeping and non-gate keeping systems? The paradoxical results of our analysis revealed that the answer to this question is not straightforward. On the one hand, we found a negative correlation between gatekeeper systems and SDM. Implying that GPs in gatekeeper systems in general, seem to be less likely to make shared decisions than GPs in a non-gatekeeping system. On the other hand, when looking in more detail at individual countries, it turns out that some countries deviate strongly from this general finding. The Netherlands, for instance, shows the highest score on SDM whereas this country has been one of the typical strong gatekeeper countries for a very long time. The same goes for the UK, a strong gatekeeper system, which shows over 80% of SDM. Other gatekeeper systems such as Spain and Denmark ended up on the other side of the distribution. This finding could potentially be related to variation between countries in for instance the level of patient trust in their GP, patient level characteristics such education level and health literacy, and beliefs about healthcare use.

We also investigated what factors GPs consider when referring to a medical specialist and how this differs between gatekeeping and non-gate keeping systems. Also, here our findings seem to nuance the negative relation between SDM and gatekeeping: it became apparent that GPs in gatekeeper systems more often take into account all kinds of patient interests, such as their preference, waiting time, costs, etc. compared to non-gatekeeping countries. In non-gatekeeping countries, the GPs’ own experiences with specialists and benchmarking information is more important. In general, it turns out that GPs apparently rely on their own experience, rather than e.g. benchmarking information.

The strength of our study lies in the large number of countries included and the statistically significant GP sample in each country and the data source similarity. Our study has also some limitations: 1) our findings are based only on GPs self-reported perception about how they do the referral, 2) no theoretical information on SDM was given to the GPs (question framed with 3 answer options: i) if they make the decision, ii) if the patient does or iii) if it is a shared decision), therefore, the results do not cover different understandings and culture around SDM, 3) we used the QUALICOPC classification of primary care systems: gatekeeping vs. non gatekeeping while other classifications (OECD) show there is also another cluster “in the middle” and a more nuanced approach to what constitutes gatekeeping can be taken and 4) the cultural bias between countries and languages and its effects on scoring “sometimes”.

Our study is one of the very few that can enrich the existing discussion (such as in BMJ) with empirical information. To our knowledge, this study is unique in the sense of providing a large international image, through the large number of countries included.

These results will aid clinicians having an overall view of the international practice. These mixed results may urge policymakers for 1) reconsidering the existing primary care mechanisms, 2) rethinking the use of ‘gatekeeper’ as a metaphor for something which is probably more a guide. The term gatekeeper seems to suggest that such GPs are mainly interested in protecting what is behind the gate and in preventing unwanted visitors from entering, rather than that they care about the preferences of people. This clearly is in sharp contrast with what this study showed. Likewise, our results will offer a debating ground for research articles for reflections like this. It seems a more nuanced and contextualized approach is needed to understand the relation between gatekeeping as a system design feature and its relation with and/or impact on shared decision making.

There is a need for further research. Our study is based on GPs’ perception, but it is also important to know how patients perceive the referral decision-making practice. Also, it is important to know what the main aspects are that GPs need to take into consideration when referring patients. International comparative research can help to understand the underlying phenomena but should be accompanied by contextualized knowledge on national situations to provide evidence for policy makers to redesign their health care system to optimize patient centeredness, efficiency and effectiveness alike.

## Supporting information

S1 Table Overview of the ethics committees in each country. (PDF)
