# Supplementary material for: Shared decision making between patient and GP about referrals from primary care: Does gatekeeping make a difference?
Source: PLoS One. 2018 Jun 11;13(6):e0198729. doi: 10.1371/journal.pone.0198729 (PMC5995363; doi:10.1371/journal.pone.0198729)
Supplement: S1 Table — (PDF) [file pone.0198729.s001.pdf]

Overview of the ethics committees in each country

Country	Ethics committee
Australia	The Australian National University (ANU) Human Research Ethics Committee.
	University of Western Sydney Human Research Ethics Committee.
	The Royal Australian College of General Practitioners (RACGP) National Research and Evaluation Ethics Committee.
Austria	Ethics committee of the Medical University of Vienna.
Belgium	University Hospital Ghent - Commission for Medical Ethics.
Bulgaria	The coordinator sent an official letter to the Ministry of Health which gave consent and support for the survey. The coordinator confirmed that there is no statutory requirement for ethical approval for this study.
Canada	10 different ethics boards*.
Cyprus	National Bioethical Committee of Cyprus.
Czech Republic	General University Hospital linked to the First Faculty of Medicine, Charles University in Prague.
Denmark	The coordinator confirmed that there is no statutory requirement for ethical approval for this study.
Estonia	The national coordinator consulted with the Ethics Review Committee on Human Research of the University of Tartu. It was confirmed that there is no statutory requirement for ethical approval for this study.
Germany	Ethics Commision of the "Landesärtzenkammer Hessen".
Hungary	National Ethical Committee.
Iceland	The Icelandic Bioethics Committee. A national committee under the Ministry of Welfare.
Ireland	Irish College of General Practitioners Research Ethics Committee – National Committee.
Italy	At Local Health Authorities level. Approval was requested from LHA Ethical Committees.
Latvia	Latvian Physicians Association Board of Certification.
Lithuania	Kauno Regionus Biomedicininu Tyrimu Etikos Komitetas.
Luxembourg	National committee of Research Ethic (CNER) in Luxembourg.
FYR Macedonia	Medical Faculty Skopje, R.Macedonia.
Malta	University of Malta Research Ethics Committee.
Netherlands	The ethics committee of VU Medisch Centrum confirmed via an official letter that the research is outside the scope of the WMO (Medical Research Involving Human Subjects Act).
New Zealand	Northern regional committee (Northern Y) for the nationally coordinated Health and Disability Ethics Committees (HDEC).
Norway	The coordinator confirmed that there is no statutory requirement for ethical approval for this study.
Poland	Bioethics approval of Jagiellonian University
Portugal	Ethical committee of Lisbon and Oporto regions; the National Commission for Health Data Safety.
Romania	Scientific Committee of CPSS.
Spain	Research Units of Primary Care of the Autonomous Community in the Basque Country. In all other Autonomous Communities, the study was approved at the Healthcare Area level.
Slovakia	The national coordinator consulted the Council of the Slovak Society of General Practice. It was confirmed that there is no statutory requirement for ethical approval for this study.
Slovenia	National medical ethics committee.
Sweden	Regional Research Ethics Committte.
Switzerland	Ethical Committee of the University of Lausanne.
Turkey	Ethical committee of Kartal Research and Education Hospital in Istanbul.
United Kingdom	University of Lincoln School of Health and Social Care Ethics Committee; National Research Ethics Service.

* See: Wong ST, Chau LW, Hogg W et al. An international cross-sectional survey on the Quality and Costs of Primary Care (QUALICO-PC): recruitment and data collection of places delivering primary care across Canada. BMC Family Practice (2015) 16:20.
